# SPINT1-AS1 Drives Cervical Cancer Progression via Repressing miR-214 Biogenesis

**DOI:** 10.3389/fcell.2021.691140

**Published:** 2021-07-19

**Authors:** Hongjuan Song, Yuan Liu, Hui Liang, Xin Jin, Liping Liu

**Affiliations:** ^1^Department of Gynecology, Xuzhou Maternal and Child Health Care Hospital, Xuzhou, China; ^2^Department of Gynecology, Xuzhou Renci Hospital, Xuzhou, China; ^3^Department of Cervical Disease, Xuzhou Maternal and Child Health Care Hospital, Xuzhou, China; ^4^Medical Department, Xuzhou Central Hospital, Xuzhou, China; ^5^Department of Research and Development, Shanghai Lichun Biotechnology Co., Ltd., Shanghai, China

**Keywords:** SPINT1-AS1, cervical cancer, progression, miR-214 biogenesis, Wnt/β-catenin signaling

## Abstract

Accumulating evidences have revealed the dysregulated expressions and critical roles of non-coding RNAs in various malignancies, including cervical cancer. Nevertheless, our knowledge about the vast majority of non-coding RNAs is still lacking. Here we identified long non-coding RNA (lncRNA) SPINT1-AS1 as a novel cervical cancer-associated lncRNA. SPINT1-AS1 was increased in cervical cancer and correlated with advanced stage and poor prognosis. SPINT1-AS1 was a direct downstream target of miR-214, a well-known tumor suppressive microRNA (miRNA) in cervical cancer. Intriguingly, SPINT1-AS1 was also found to repress miR-214 biogenesis via binding DNM3OS, the primary transcript of miR-214. The interaction between SPINT1-AS1 and DNM3OS repressed the binding of DROSHA and DGCR8 to DNM3OS, blocked DNM3OS cleavage, and therefore repressed mature miR-214 biogenesis. The expression of SPINT1-AS1 was significantly negatively correlated with miR-214 in cervical cancer tissues, supporting the reciprocal repression between SPINT1-AS1 and miR-214 *in vivo*. Through downregulating mature miR-214 level, SPINT1-AS1 upregulated the expression of β-catenin, a target of miR-214. Thus, SPINT1-AS1 further activated Wnt/β-catenin signaling in cervical cancer. Functionally, SPINT1-AS1 drove cervical cancer cellular proliferation, migration, and invasion *in vitro*, and also tumorigenesis *in vivo*. Deletion of the region mediating the interaction between SPINT1-AS1 and DNM3OS, overexpression of miR-214, and inhibition of Wnt/β-catenin signaling all reversed the roles of SPINT1-AS1 in cervical cancer. Collectively, these findings identified SPINT1-AS1 as a novel cervical cancer-associated oncogenic lncRNA which represses miR-214 biogenesis and activates Wnt/β-catenin signaling, highlighting its potential as prognostic biomarker and therapeutic target for cervical cancer.

## Introduction

Cervical cancer is the fourth most common and fourth leading cause of cancer-associated death in women, with 604,127 new cervical cancer cases and 341,831 new cervical cancer deaths in 2020 worldwide (Sung et al., 2021). Although vaccination and early diagnosis have decreased the incidence and mortality rates of cervical cancer, those patients with advanced or recurrent cervical cancers have limited therapeutic options (Wolford and Tewari, 2018). Therefore, further studies are urgently needed to reveal pathogenic mechanisms of cervical cancer and develop more effective targeted therapies.

Many studies are struggling to reveal the molecular mechanisms underlying cervical cancer (Cerasuolo et al., 2020; Lourenco de Freitas et al., 2020). Apart from the canonical oncogenes and tumor suppressors involved in cervical cancer, high throughput transcriptomic sequencings have identified more non-coding RNAs (ncRNAs) with dysregulated expression in cervical cancer (Berger et al., 2018). microRNAs (miRNAs) is one class of ncRNAs with 19∼23 nucleotides in length (Moro et al., 2019; Wang et al., 2019). The expressions and roles of miRNAs have been intensively investigated in cervical cancer (Chen et al., 2019; Zheng et al., 2019). Several previous reports, including ours, have revealed miR-214 as a down-regulated and tumor suppressive miRNA in cervical cancer (Song et al., 2019; Peng et al., 2020; Tornesello et al., 2020). However, the factors responsible for the downregulation of miR-214 in cervical cancer are still unknown. Canonically, primary miRNAs (pri-miRNAs) are transcribed, which are further cleaved by Microprocessor comprised of RNase III enzyme DROSHA and DGCR8 to yield the pre-miRNAs. The pre-miRNAs are exported to cytoplasm where they were cleaved by DICER to generate mature miRNAs (Spadotto et al., 2020; Uebbing et al., 2021). miR-214 is located in the DNM3OS transcript unit which gives rise to mature miR-214 (Qin et al., 2012; Savary et al., 2019).

Apart from miRNAs, long non-coding RNAs (lncRNAs) is another class of ncRNAs which are over 200 nucleotides in length (Zhu et al., 2016; Esposito et al., 2019; Luo et al., 2019; Budkova et al., 2020; Zhang et al., 2020). Similar to miRNAs, several lncRNAs were shown to be abnormally expressed and play oncogenic or tumor suppressive roles in cervical cancer (Han et al., 2020; Wang et al., 2020). Deep-sequencings of human transcriptome identified over 58,000 lncRNAs (Iyer et al., 2015). Until now, only a part of lncRNAs were investigated. Potential roles of other lncRNAs need further investigation.

Unlike miRNAs, the molecular mechanisms of lncRNAs are complex (Ernst et al., 2018; Ghafouri-Fard et al., 2021). Canonically, miRNAs are loaded into AGO2 to yield the RNA-induced silencing complex (RISC), which represses miRNAs’ targets expression (Yuan et al., 2011; Quevillon Huberdeau and Simard, 2019). For lncRNAs, one mechanism is competitively binding miRNAs and further relieving the suppressive effects of miRNAs on their targets (Song et al., 2019; Li et al., 2020). Another mechanism of lncRNAs is directly binding proteins and further changes the stability and function of the interacted proteins (Li et al., 2017; Daneshvar et al., 2020; Zhang et al., 2020). lncRNAs were also reported to directly interact with mRNAs and further change the stability and/or translation of the interacted mRNAs (Mo et al., 2020).

Given the tumor suppressive roles of miR-214 in cervical cancer, we hypothesized that the lncRNAs that modulate miR-214 might play critical roles in cervical cancer. Therefore, we screened lncRNAs which have the potential to bind miR-214. Furthermore, the expressions of these lncRNAs in cervical cancer were analyzed using publicly available datasets. Combining these analyses, we identified a novel cervical cancer-associated lncRNA SPINT1-AS1. The expression and clinical relevance of SPINT1-AS1 in cervical cancer were detected. Gain and loss of function assays were carried out to elucidate the potential roles of SPINT1-AS1 in cervical cancer. Moreover, the roles of miR-214 in modulating SPINT1-AS1 and the roles of SPINT1-AS1 in modulating miR-214 and its downstream targets were investigated in detail.

## Materials and Methods

### Bioinformatics Analysis

The lncRNAs interact with miR-214 were analyzed using The Encyclopedia of RNA Interactomes (ENCORI)^1^ supported by Ago crosslinking-immunoprecipitation and high-throughput sequencing (CLIP-seq) data. The expressions of lncRNAs in cervical cancer tissues and normal cervical tissues from The Cancer Genome Atlas (TCGA) and the Genotype-Tissue Expression (GTEx) projects were analyzed using Gene Expression Profiling Interactive Analysis (GEPIA)^2^. Coding potential of SPINT1-AS1 was calculated using the Coding Potential Assessment Tool (CPAT)^3^ and the Coding Potential Calculator (CPC)^4^. Subcellular localization of SPINT1-AS1 was predicted using lncLocator^5^. SPINT1-AS1 and DNM3OS interaction was predicted by IntaRNA^6^.

### Cell Culture

Human cervical cancer cell lines HeLa and SiHa were purchased from the American Type Culture Collection (Manassas, VA, United States). These cells were cultured in Eagle’s Minimum Essential Medium (MEM) (Invitrogen, Carlsbad, CA, United States) added with 10% fetal bovine serum (Invitrogen) at 37°C with 5% CO_2_.

### Tissue Specimens

Ninety-two pairs of cervical cancer tissues and adjacent normal cervical tissues were collected from cervical cancer patients with written informed consent at Xuzhou Maternity and Child Health Care Hospital (Xuzhou, China). All tissues were confirmed by pathological examination. The clinicopathological characteristics of these 92 cases are shown in Table 1. This study was conducted following the principles of Declaration of Helsinki. The Ethics Committee of Xuzhou Maternity and Child Health Care Hospital approved this study.

**TABLE 1:** Correlation between SPINT1-AS1 expression and clinicopathological characteristics in 92 cases of cervical cancer.

**Characteristics**	**N**	**SPINT1-AS1 expression**		***P-*value**
		**Low**	**High**	
Age (years)				0.524
≥45	55	29	26	
<45	37	17	20	
Histology				0.456
Squamous	71	34	37	
Adenocarcinoma	21	12	9	
Tumor size (cm)				0.031
≥4	34	12	22	
<4	58	34	24	
FIGO stage				0.018
I	57	34	23	
II	35	12	23	
Lymph node metastasis				0.026
Positive	30	10	20	
Negative	62	36	26	

**P-values were calculated by Pearson chi-square test.**

### RNA Extraction and Quantitative Real−Time Polymerase Chain Reaction (qRT-PCR)

Total RNA was extracted from cervical cancer tissues and cells using the TRIzol reagent (Invitrogen). The RNA was used to carry out reverse transcription with the PrimeScript^TM^ II 1st Strand cDNA Synthesis Kit (Takara, Dalian, China) to acquire first-stand cDNA. To quantitate lncRNAs and mRNAs expression, qRT−PCR was conducted using TB Green^®^ Premix Ex Taq^TM^ II (Takara) on 7500 Real-Time PCR System (Applied Biosystems, Foster City, CA, United States) with the primers 5′-AGCCAGACAGACGGACAGG-3′ (forward) and 5′-GCAGCACAAACTTCTTTACATC-3′ (reverse) for SPINT1-AS1, 5′-TGTGAGTTTTCTGTTACGC-3′ (forward) and 5′-TACAATCAGCCTGTTTTCC-3′ (reverse) for DNM3OS, 5′-CTTCCCCTACCCTCTCAA-3′ (forward) and 5′-CGATTTCTTCCTCATCTTCT-3′ (reverse) for c-Myc, 5′-ACAACTTCCTGTCCTACTACCG-3′ (forward) and 5′-TCCTCTTCCTCCTCCTCG-3′ (reverse) for cyclin D1, 5′-GGTCTCCTCTGACTTCAACA-3′ (forward) and 5′-GTGAGGGTCTCTCTCTTCCT-3′ (reverse) for GAPDH. To quantitate miRNAs expression, qRT−PCR was conducted using the TaqMan^TM^ Advanced miRNA Assay (Applied Biosystems) on 7500 Real-Time PCR System. GAPDH was used as endogenous control to quantitate lncRNAs and mRNAs expression. U6 was used as endogenous control to quantitate miRNAs expression. The comparative Ct method was used to calculate the expression of RNAs.

### Isolation of Cytoplasmic and Nuclear RNA

Cytoplasmic and nuclear RNA was isolated from HeLa cells by the Cytoplasmic and Nuclear RNA Purification Kit (Norgen, Belmont, CA, United States) as we previously described (Song et al., 2019). The expression levels of SPINT1-AS1, GAPDH, and U6 in cytoplasm and nucleus were detected by qRT-PCR.

### Plasmids Construction and Transfection

SPINT1-AS1 containing the predicted miR-214 binding site were PCR-amplified using the Platinum^®^ *Pfx* DNA Polymerase (Invitrogen) with the primers 5′-CCGCTCGAGTTCCGAGGGTGCTGGTG- 3′ (forward) and 5′-GCTCTAGAGTAGGGGGGATTCTGGGAGTAG-3′ (reverse). Next, the PCR products were inserted into the Xho I and Xba I sites of the pmirGLO Dual-Luciferase miRNA Target Expression Vector (Promega, Madison, WI, United States) to construct pmirGLO-SPINT1-AS1. pmirGLO-SPINT1-AS1 with miR-214 binding site mutation (pmirGLO-SPINT1-AS1-mut) was constructed using the Fast Mutagenesis System (TransGen Biotech, Beijing, China) with the primers 5′-AGGAAAAGGGAAGGGGGACGACTCTTCCAGCCAC-3′ (forward) and 5′-GTCGTCCCCCTTCCCTTTTCCTTTA GAGTCTCTC-3′ (reverse). CTNNB1 3′-UTR containing the predicted miR-214 binding site were PCR-amplified using the Platinum^®^ *Pfx* DNA Polymerase (Invitrogen) with the primers 5′-CGAGCTCGAGTGGTTTAGGCTATTTG-3′ (forward) and 5′-GCTCTAGACATTTTCTCTTGAAGCATCG-3′ (reverse). Next, the PCR products were inserted into the Sac I and Xba I sites of pmirGLO to generate pmirGLO-CTNNB1.

SPINT1-AS1 full-length sequences were PCR-amplified by the Platinum^®^ *Pfx* DNA Polymerase (Invitrogen) with the primers 5′-CCCAAGCTTAGCGCGGGCCTCTGGGTT-3′ (forward) and 5′-GGAATTCACCAGCTTGAAACAATAAGCATTTA-3′ (reverse). 5′ 70 nucleotides deleted SPINT1-AS1 sequences were PCR-amplified by the Platinum^®^ *Pfx* DNA Polymerase (Invitrogen) with the primers 5′-CCCAAGCTTCCTGATCAGCCCGGGAGAC-3′ (forward) and 5′ -GGAATTCACCAGCTTGAAACAATAAGCATTTA-3′ (reverse). Next, the PCR products were inserted into the Hind III and EcoR I sites of the pcDNA^TM^3.1(+) gene expression vector (Invitrogen) to generate pcDNA3.1-SPINT1-AS1 and pcDNA3.1-SPINT1-AS1-del, respectively. Moreover, the PCR products were also inserted into the Hind III and EcoR I sites of the pSPT19 transcription vector (Roche, Mannheim, Germany) to generate pSPT19-SPINT1-AS1 and pSPT19-SPINT1-AS1-del, respectively.

The MS2-12 × fragment was PCR-amplified by the Platinum^®^ *Pfx* DNA Polymerase (Invitrogen) from pSL-MS2-12 × (Addgene, Watertown, MA, United States) with the primers 5′-ATGATATCCCGGGCCCTATATATGGATC-3′ (forward) and 5′-CCGCTCGAGTATCGATCGCGCGCAGATCTA-3′ (reverse). Next, the PCR products were inserted into the EcoR V and Xho I sites of pcDNA3.1, pcDNA3.1-SPINT1-AS1, or pcDNA3.1-SPINT1-AS1-del, to construct pcDNA3.1-MS2, pcDNA3.1-MS2-SPINT1-AS1, or pcDNA3.1-MS2-SPINT1-AS1-del, respectively.

miR-214 mimics, inhibitors, and their respective negative controls (NC) were obtained from GenePharma (Shanghai, China). The transfection and co-transfection of miRNAs and plasmids were performed using the Lipofectamine 3000 (Invitrogen).

### Stable Cell Lines Construction

To stably overexpress full-length SPINT1-AS1 or 5′ 70 nucleotides deleted SPINT1-AS1 in cervical cancer cells, pcDNA^TM^3.1(+), pcDNA3.1-SPINT1-AS1, and pcDNA3.1-SPINT1-AS1-del were transfected into HeLa cells. pcDNA^TM^3.1(+) and pcDNA3.1-SPINT1-AS1 were transfected into SiHa cells. After culture for another 48 h, the cells were selected with neomycin for 4 weeks. To obtain SPINT1-AS1 and miR-214 concurrently overexpressed HeLa cells, SPINT1-AS1 stably overexpressed HeLa cells were infected with miR-214 overexpression lentiviruses (FulenGen, Guangzhou, China). After culture for another 96 h, the cells were selected with puromycin and neomycin for 4 weeks. Two pairs of cDNA oligonucleotides targeting SPINT1-AS1 were designed and generated by GenePharma. After annealing, double-strand oligonucleotides were inserted into the GenePharma Supersilencing Vector pLV6(EF-1a/Puro) to generate lentivirus shRNA targeting SPINT1-AS1. A scrambled non-targeting lentivirus shRNA was used as negative control (NC). HeLa and SiHa cells were infected with these lentivirus shRNAs. After culture for another 96 h, the cells were selected with puromycin for 4 weeks. The shRNA sequences were as follows: 5′-GATCCGGAGGAGACACACCTGATCAGTTCAAGAGACTGA TCAGGTGTGTCTCCTCCTTTTTTG-3′ (forward) and 5′-AATTCAAAAAAGGAGGAGACACACCTGATCAGTCTCTT GAACTGATCAGGTGTGTCTCCTCCG-3′ (reverse) for sh RNA-SPINT1-AS1-1, 5′-GATCCGACCGCTAGGGAGCTCAA GTATTCAAGAGATACTTGAGCTCCCTAGCGGTCTTTTTT G-3′ (forward) and 5′-AATTCAAAAAAGACCGCTAGGG AGCTCAAGTATCTCTTGAATACTTGAGCTCCCTAGCGGT CG-3′ (reverse) for shRNA-SPINT1-AS1-2, 5′-GATCCGT TCTCCGAACGTGTCACGTTTCAAGAGAACGTGACACGTT CGGAGAACTTTTTTG-3′ (forward) and 5′-AATTCAAAA AAGTTCTCCGAACGTGTCACGTTCTCTTGAAACGTGACA CGTTCGGAGAACG-3′ (reverse) for shRNA-NC.

### Dual Luciferase Reporter Assay

pmirGLO, pmirGLO-SPINT1-AS1, or pmirGLO-SPINT1-AS1 was co-transfected with miR-214 mimics or miR-NC into HeLa cells. pmirGLO, pmirGLO-SPINT1-AS1, or pmirGLO-SPINT1-AS1 was co-transfected with miR-214 inhibitors or inh-NC into HeLa cells. pmirGLO or pmirGLO-CTNNB1 was transfected into HeLa cells with SPINT1-AS1 or SPINT1-AS1-del stable overexpression. pmirGLO or pmirGLO-CTNNB1 was transfected into HeLa cells with SPINT1-AS1 stable silencing. β-catenin reporter TOPFlash (Addgene) or control reporter FOPFlash (Addgene) was co-transfected with pRL-TK (Promega) into HeLa cells with SPINT1-AS1 or SPINT1-AS1-del stable overexpression. pRL-TK was used as an internal control reporter which encodes Renilla luciferase. TOPFlash or FOPFlash was co-transfected with pRL-TK into HeLa cells with SPINT1-AS1 stable silencing. After culture for another 48 h, firefly luciferase and Renilla luciferase activities were measured using the Dual-Luciferase^®^ Reporter Assay System (Promega).

### RNA Affinity Pulldown Assays

RNA affinity pulldown assays were performed as previously described (Yuan et al., 2017). Briefly, biotin-labeled SPINT1-AS1 and SPINT1-AS1-del were, respectively, *in vitro* transcribed from pSPT19-SPINT1-AS1 and pSPT19-SPINT1-AS1-del with the Biotin RNA Labeling Mix (Roche) and T7 RNA polymerase (Roche). After RNase-free DNase I (Roche) treatment, the *in vitro* transcribed transcripts were purified using the RNeasy Mini Kit (Qiagen, Valencia, CA, United States). Three μg of purified transcripts were incubated with 1 mg of whole-cell lysates from HeLa cells for 1 h at 25°C. Next, the complexes were isolated using streptavidin agarose beads (Invitrogen). The RNA present in the pulldown material was measured by qRT-PCR.

### RNA Immunoprecipitation (RIP) Assays

pcDNA3.1-MS2, pcDNA3.1-MS2-SPINT1-AS1, or pcDNA3.1-MS2-SPINT1-AS1-del was co-transfected with pMS2-GFP (Addgene) into HeLa cells. After culture for another 48 h, cells were used to conduct RIP assays using the Magna RIP RNA-Binding Protein Immunoprecipitation Kit (Millipore, Billerica, MA, United States) and a GFP antibody (5 μg per reaction; 11814460001, Roche). For anti-DROSHA, anti-DGCR8 RIP, HeLa cells with SPINT1-AS1 or SPINT-AS1-del stable overexpression and HeLa cells with SPINT1-AS1 stable silencing were used to conduct RIP assays using a DROSHA antibody (5 μg per reaction; #3410, Cell Signaling Technology, Danvers, MA, United States), a DGCR8 antibody (5 μg per reaction; ab191875, Abcam, Hong Kong, China), and the Magna RIP RNA-Binding Protein Immunoprecipitation Kit.

### Biotinylated Anti-sense Oligo Probes Capture Assays

Biotinylated anti-sense oligo probes against SPINT1-AS1 were designed and generated by Biosearch Probe Designer. The probe sequences were as follows: 1, 5′-tcggaacccagaggcccgcg-3′; 2, 5′-cgcctcctccaaagtctccc-3′; 3, 5′-ctggagggcgcgtgggaggg-3′; 4, 5′-ctttgttctgcgctcgtgcg-3′; 5, 5′-ttacatcacagctggctctg-3′; 6, 5′-ttcccttttcctttagagtc-3′; 7, 5′-gcttacttatcacatggcac-3′; 8, 5′-actcacaagggtgttggcag-3′, 9, 5′-gctcagggactgcagtcaag-3′; 10, 5′-ttttttttttaccagcttga-3′. These probes and the Magna ChIRP^TM^ RNA Interactome Kits (Millipore) were used to capture SPINT1-AS1 and its interacted RNAs in HeLa cells. The captured RNAs were measured by qRT-PCR.

### Western Blot

Western blot was carried out as we previously described (Song et al., 2019). Briefly, total proteins were extracted from HeLa cells with SPINT1-AS1 or SPINT-AS1-del stable overexpression and HeLa cells with SPINT1-AS1 stable silencing using RIPA lysis buffer (Beyotime, Shanghai, China). Total protein was separated by 10% sodium dodecyl sulfate-polyacrylamide gel electrophoresis, followed by being transferred to polyvinylidene difluoride membrane (Millipore). After block using 5% non-fat milk at room temperature for 2 h, the membranes were incubated with primary antibodies against β-catenin (1:1,000, #8480, Cell Signaling Technology) or GAPDH (1:1,000, #97166, Cell Signaling Technology) overnight at 4°C. After further incubation with IRDye 700-conjugated goat anti-mouse IgG or IRDye 800-conjugated goat anti-rabbit IgG second antibodies (Invitrogen), the membranes were scanned on the Odyssey infrared scanner (Li-Cor, Lincoln, NE, United States). GAPDH was used as endogenous control for to quantitate β-catenin protein levels.

### Cell Proliferation, Migration, and Invasion Assays

Glo cell viability and 5-Ethynyl-2′-deoxyuridine (EdU) staining experiments were performed to evaluate cell proliferation as we previously described (Song et al., 2019). Glo cell viability experiment was carried out using the CellTiter-Glo^®^ Luminescent Cell Viability Assay (Promega). EdU staining experiment was carried out using the EdU kit (RiboBio, Guangzhou, China). Transwell migration assay was performed to evaluate cell migration as we previously described (Song et al., 2019). Transwell invasion assay was performed to evaluate cell invasion as we previously described (Song et al., 2019).

### Xenograft Assay in Mice

Five-week-old female BALB/c-nu/nu nude mice were bred in the pathogen-free condition. Animal experiments were approved by the Ethics Committee of the Xuzhou Maternity and Child Health Care Hospital. 2 × 10^6^ HeLa cells with SPINT1-AS1 or SPINT-AS1-del stable overexpression were subcutaneously injected into nude mice. Subcutaneous tumor volumes were measured every 4 days using a caliper. The volumes were calculated using the equation V = 0.5 × a × b^2^ (a, long axes; b, short axes). On the 20th day after injection, subcutaneous tumors were resected and weighed. For the evaluation of Ki67, cleaved caspase-3, and β-catenin expressions in subcutaneous tumors, immunohistochemistry (IHC) staining was performed using the primary antibodies against Ki67 (1:200, ab15580, Abcam), cleaved caspase-3 (1:200, #9661, Cell Signaling Technology), or β-catenin (1:100, #8480, Cell Signaling Technology) as we previously described (Song et al., 2019).

### Statistical Analysis

Statistical analyses were carried out using GraphPad Prism 6 software. For comparisons, Student’s *t*-test, one-way analysis of variance (ANOVA) followed by Dunnett’s multiple comparisons test, non-parametric Spearman correlation analysis, Wilcoxon matched-pairs signed rank test, log-rank test, and Pearson chi-square test were performed as described in figure and table legends. *P* < 0.05 was considered as statistically significant.

## Results

### SPINT1-AS1 Was a Downstream Target of miR-214

**FIGURE 1:**
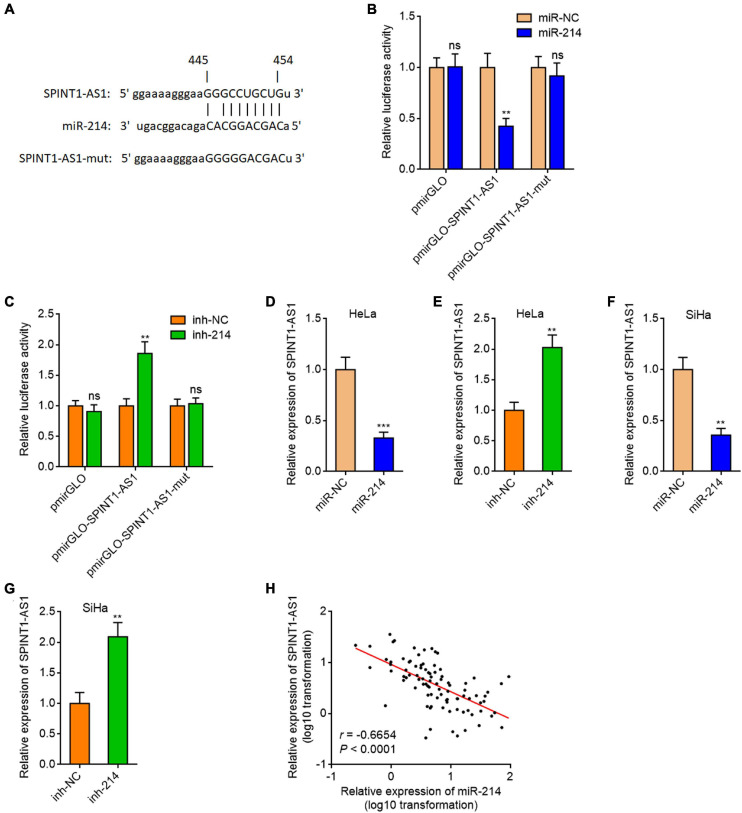
SPINT1-AS1 was a target of miR-214 in cervical cancer. **(A)** Schematic diagram of the predicted miR-214 binding site on SPINT1-AS1. **(B)** Dual luciferase reporter assays in HeLa cells after co-transfection of luciferase reporter containing nothing, SPINT1-AS1, or mutated SPINT1-AS1 with miR-214 mimics or miRNA negative control (miR-NC). Data are shown as the relative ratio of firefly luciferase activity to renilla luciferase activity. **(C)** Dual luciferase reporter assays in HeLa cells after co-transfection of luciferase reporter containing nothing, SPINT1-AS1, or mutated SPINT1-AS1 with miR-214 inhibitors or inhibitor negative control (inh-NC). Data are shown as the relative ratio of firefly luciferase activity to renilla luciferase activity. **(D)** SPINT1-AS1 expression in HeLa cells after transfection of miR-214 mimics or miR-NC was detected by qRT-PCR. **(E)** SPINT1-AS1 expression in HeLa cells after transfection of miR-214 inhibitors or inh-NC was detected by qRT-PCR. **(F)** SPINT1-AS1 expression in SiHa cells after transfection of miR-214 mimics or miR-NC was detected by qRT-PCR. **(G)** SPINT1-AS1 expression in SiHa cells after transfection of miR-214 inhibitors or inh-NC was detected by qRT-PCR. For **(B–G)**, results are shown as mean ± SD based on three independent experiments. **P < 0.01, ***P < 0.001, ns, not significant, by Student’s *t*-test. **(H)** The correlation between SPINT1-AS1 and miR-214 expression levels in 92 cervical cancer tissues. *r* = –0.6654, *P* < 0.0001 by non-parametric Spearman correlation analysis.

In our previous study, we predicted 209 lncRNAs which may interact with miR-214 supported by Ago CLIP-seq (crosslinking-immunoprecipitation and high-throughput sequencing) data using ENCORI (The Encyclopedia of RNA Interactomes) (see text footnote 1) (Song et al., 2019). Several reports, including ours, have shown that miR-214 was downregulated in cervical cancer (Yang et al., 2018; Song et al., 2019; Peng et al., 2020). Thus, we hypothesized that the lncRNAs which regulate miR-214 or be regulated by miR-214 should be upregulated in cervical cancer. The expression of candidate lncRNAs in cervical cancer tissues and normal cervical tissues from The Cancer Genome Atlas (TCGA) and the Genotype-Tissue Expression (GTEx) projects were analyzed using GEPIA (Gene Expression Profiling Interactive Analysis) (see text footnote 2). Notably, a candidate lncRNAs SPINT1-AS1 was predicted to be significantly upregulated in cervical cancer tissues (Figure 1A and Supplementary Figure 1A). To investigate whether SPINT1-AS1 is a miR-214 target, SPINT1-AS1 sequences containing the predicted miR-214 binding site were inserted into the pmirGLO Dual-Luciferase miRNA Target Expression Vector. Dual luciferase reporter assays demonstrated that miR-214 remarkably reduced the luciferase activity of pmirGLO reporter containing SPINT1-AS1, but not the empty pmirGLO reporter (Figure 1B). Conversely, inhibition of miR-214 remarkably increased the luciferase activity of pmirGLO reporter containing SPINT1-AS1 (Figure 1C). Furthermore, miR-214 binding site-mutated SPINT1-AS1 was inserted into the pmirGLO reporter. Dual luciferase reporter assays demonstrated that the mutation of miR-214 binding site on SPINT1-AS1 blocked the influences of miR-214 on pmirGLO reporter containing SPINT1-AS1 (Figures 1B,C), suggesting that the predicted miR-214 binding site was responsible for the regulation of SPINT1-AS1 by miR-214. To further test whether SPINT1-AS1 is a target of miR-214 in cervical cancer, SPINT1-AS1 expression in HeLa cells was measured after transient transfection of miR-214 mimics or inhibitors. The results demonstrated that miR-214 reduced SPINT1-AS1 expression and while inhibition of miR-214 increased SPINT1-AS1 expression (Figures 1D,E). The same results were also acquired in SiHa cells (Figures 1F,G). We collected 92 cervical cancer tissues to detect the expression of SPINT1-AS1 and miR-214. The results demonstrated that SPINT1-AS1 expression was significantly negatively correlated with miR-214 expression in cervical cancer tissues (Figure 1H), supporting SPINT1-AS1 as a target of miR-214 *in vivo*. Two *in silico* tools, the Coding Potential Assessment Tool (CPAT) (see text footnote 3) and the Coding Potential Calculator (CPC) (see text footnote 4) both indicated SPINT1-AS1 as non-coding RNA (Supplementary Figures 1B,C). *In silico* tool lncLocator (see text footnote 5) predicted that SPINT1-AS1 was localized in both cytoplasm and nucleus (Supplementary Figure 1D). Subcellular fraction followed by qRT-PCR further confirmed the localization of SPINT1-AS1 in both cytoplasm and nucleus (Supplementary Figure 1E).

### SPINT1-AS1 Repressed miR-214 Biogenesis via Binding DNM3OS

**FIGURE 2:**
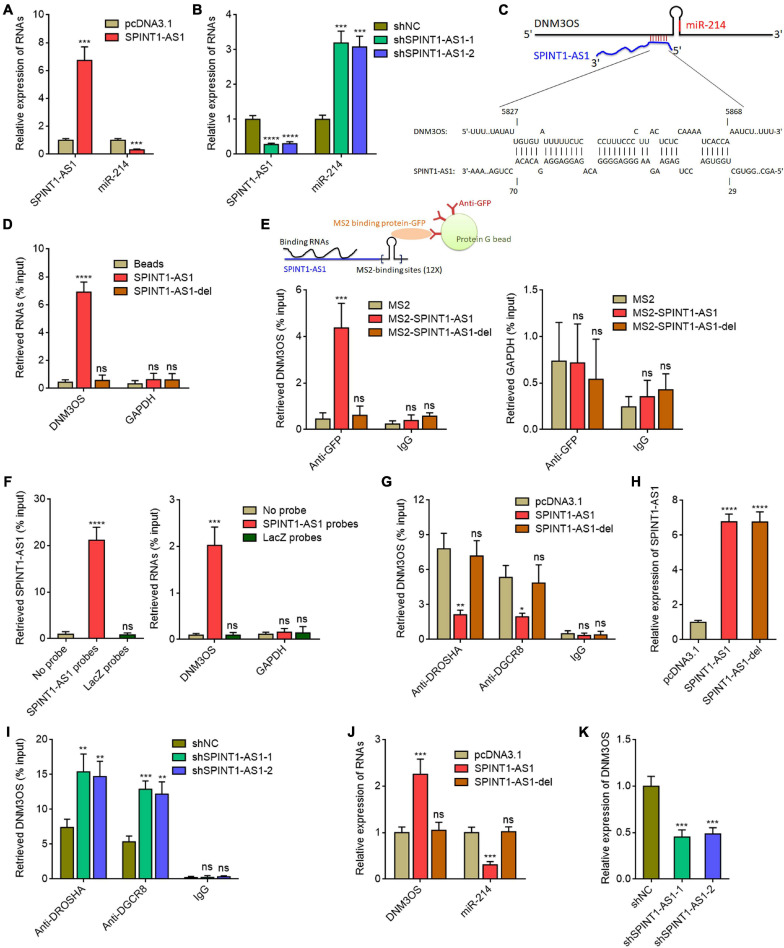
SPINT1-AS1 repressed miR-214 biogenesis via binding DNM3OS. **(A)** SPINT1-AS1 and miR-214 expressions in HeLa cells with SPINT1-AS1 stable overexpression were detected by qRT-PCR. **(B)** SPINT1-AS1 and miR-214 expressions in HeLa cells with SPINT1-AS1 stable silencing were detected by qRT-PCR. **(C)** Schematic diagram of the predicted interaction between DNM3OS and SPINT1-AS1. **(D)** RNA affinity pulldown assays using *in vitro* transcribed biotinylated SPINT1-AS1 or 5′ 70 nucleotides deleted SPINT1-AS1 (SPINT1-AS1-del) were conducted to retrieve the RNAs interacted with SPINT1-AS1. The retrieved RNAs were detected by qRT-PCR. **(E)** MS2 vector-based RIP assays were conducted in HeLa cells to retrieve the RNAs interacted with SPINT1-AS1. The retrieved RNAs were detected by qRT-PCR. **(F)** Biotinylated anti-sense oligo probes against SPINT1-AS1 were used to capture endogenous SPINT1-AS1 and the RNAs interacted with SPINT1-AS1 in HeLa cells. The retrieved RNAs were detected by qRT-PCR. **(G)** RIP assays using DROSHA and DGCR8 antibodies were performed in HeLa cells with SPINT1-AS1 or SPINT1-AS1-del stable overexpression. The retrieved RNA was measured by qRT-PCR. **(H)** SPINT1-AS1 expression in HeLa cells with SPINT1-AS1 or SPINT1-AS1-del stable overexpression was detected by qRT-PCR. **(I)** RIP assays using DROSHA and DGCR8 antibodies were performed in HeLa cells with SPINT1-AS1 stable silencing. **(J)** DNM3OS and miR-214 expressions in HeLa cells with SPINT1-AS1 or SPINT1-AS1-del stable overexpression were detected by qRT-PCR. **(K)** DNM3OS expression in HeLa cells with SPINT1-AS1 stable silencing was detected by qRT-PCR. Results are shown as mean ± SD based on three independent experiments. *P < 0.05, **P < 0.01, ***P < 0.001, ****P < 0.0001, ns, not significant, by Student’s *t*-test **(A)** or one-way ANOVA followed by Dunnett’s multiple comparisons test **(B,D–K)**.

Given the significantly inverse correlation (*r* = −0.6654) between SPINT1-AS1 expression and miR-214 expression in cervical cancer tissues, we investigated whether SPINT1-AS1 also modulates miR-214 expression. We constructed SPINT1-AS1 stably overexpressed HeLa cells using SPINT1-AS1 overexpression plasmid (Figure 2A). Intriguingly, miR-214 was found to be significantly downregulated in SPINT1-AS1 overexpressed HeLa cells (Figure 2A). Ectopic expression of SPINT1-AS1 also reduced miR-214 expression in SiHa cells (Supplementary Figure 2A). SPINT1-AS1 stably silenced HeLa cells were constructed using two independent SPINT1-AS1 specific shRNA lentiviruses (Figure 2B). miR-214 was significantly upregulated in SPINT1-AS1 silenced HeLa cells (Figure 2B). SPINT1-AS1 silencing also increased miR-214 expression in SiHa cells (Supplementary Figure 2B). *In silico* RNA-RNA interaction tool IntaRNA (see text footnote 6) predicted an interaction region between 29 and 70 nucleotides of SPINT1-AS1 and 5827-5868 nucleotides of DNM3OS (Figure 2C). To investigate whether SPINT1-AS1 interacts with DNM3OS, RNA affinity pulldown assays with *in vitro* transcribed biotinylated SPINT1-AS1 or 5′ 70 nucleotides deleted SPINT1-AS1 (SPINT1-AS1-del) were conducted to retrieve the RNAs interacted with SPINT1-AS1. The results demonstrated that DNM3OS was specifically retrieved by full-length SPINT1-AS1, but not by 5′ 70 nucleotides deleted SPINT1-AS1 (Figure 2D). MS2 vector-based RIP assays further demonstrated that SPINT1-AS1 specifically interacted with DNM3OS, which was abolished by 5′ 70 nucleotides deletion (Figure 2E). To further detect endogenous interaction between SPINT1-AS1 and DNM3OS, biotinylated anti-sense oligo probes against SPINT1-AS1 were used to capture endogenous SPINT1-AS1 and the RNAs interacted with SPINT1-AS1. The results demonstrated that SPINT1-AS1 was successfully retrieved by SPINT1-AS1 probes (Figure 2F). Moreover, DNM3OS was also retrieved by SPINT1-AS1 probes (Figure 2F), supporting the endogenous interaction between SPINT1-AS1 and DNM3OS. DNM3OS was bound and processed by DROSHA and DGCR8 to generate pre-miR-214 and lastly miR-214 (Qin et al., 2012; Savary et al., 2019). To investigate whether the interaction between SPINT1-AS1 and DNM3OS modulates the processing of DNM3OS by DROSHA and DGCR8, we first studied the effects of SPINT1-AS1 on the binding between SPINT1-AS1 and DROSHA, DGCR8. RIP assays using DROSHA and DGCR8 antibodies demonstrated that overexpression of SPINT1-AS1 inhibited the binding of DNM3OS to DROSHA and DGCR8 (Figure 2G). Furthermore, 5′ 70 nucleotides deleted SPINT1-AS1 was also stably overexpressed in HeLa cells with similar overexpression efficiency to full length SPINT1-AS1 (Figure 2H). RIP assays demonstrated that the deletion of DNM3OS binding sites on SPINT1-AS1 abolished the effects of SPINT1-AS1 on the binding of DNM3OS to DROSHA and DGCR8 (Figure 2G). RIP assays also demonstrated that SPINT1-AS1 silencing promoted the binding of DNM3OS to DROSHA and DGCR8 (Figure 2I). Consistent with the repressive roles of SPINT1-AS1 on the binding of DNM3OS to DROSHA and DGCR8, ectopic expression of SPINT1-AS1 upregulated DNM3OS expression, which was reversed by the deletion of DNM3OS binding site on SPINT1-AS1 (Figure 2J). Similarly, the repressive roles of DNM3OS on mature miR-214 were also abolished by the deletion of DNM3OS binding site on SPINT1-AS1 (Figure 2J). Conversely, SPINT1-AS1 silencing downregulated DNM3OS expression (Figure 2K). In SiHa cells, ectopic expression of SPINT1-AS1 also upregulated and while SPINT1-AS1 silencing also repressed DNM3OS expression (Supplementary Figures 2C,D). *MIR3120* is located in the anti-sense stand of *MIR214*. *MIR199A2* is located in the intron region of *DNM3OS*. Our results showed that neither SPINT1-AS1 overexpression nor SPINT1-AS1 silencing changed miR-3120 and miR-199a expression (Supplementary Figures 2E,F), supporting that the modulation of miR-214 by SPINT1-AS1 is dependent on DNM3OS. Collectively, these results suggested that SPINT1-AS1 repressed miR-214 biogenesis via directly binding DNM3OS.

### Increased Expression of SPINT1-AS1 Was Correlated With Advanced Stage and Poor Prognosis in Cervical Cancer

**FIGURE 3:**
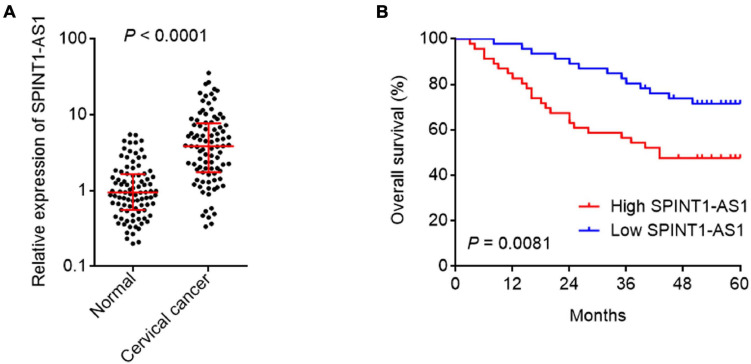
SPINT1-AS1 was increased in cervical cancer tissues and associated with poor prognosis of cervical cancer patients. **(A)** SPINT1-AS1 expression in 92 pairs of cervical cancer tissues and adjacent normal cervical tissues was detected by qRT-PCR. Results are shown as median with interquartile range. *P* < 0.0001 by Wilcoxon matched-pairs signed rank test. **(B)** Kaplan-Meier analysis of the correlation between SPINT1-AS1 expression level and overall survival of these 92 cervical cancer cases. The median expression level of SPINT1-AS1 was used as the cutoff. *P* = 0.0081 by log-rank test.

To evaluate the clinical relevance of SPINT1-AS1 in cervical cancer, SPINT1-AS1 expression in 92 pairs of cervical cancer tissues and matched adjacent normal cervical tissues was measured. The results indicated that SPINT1-AS1 was remarkably increased in cervical cancer tissues compared with normal tissues (Figure 3A). Next, the correlation between SPINT1-AS1 expression and clinicopathological characteristics in these 92 cases was analyzed. The results presented that increased expression of SPINT1-AS1 was correlated with large tumor size, advanced FIGO stage, and lymph node metastasis (Table 1). Kaplan-Meier analyses in these 92 cases demonstrated that high expression of SPINT1-AS1 was correlated with worse overall survival (Figure 3B).

### SPINT1-AS1 Drove Cervical Cancer Cellular Proliferation, Migration, and Invasion *in vitro*

**FIGURE 4:**
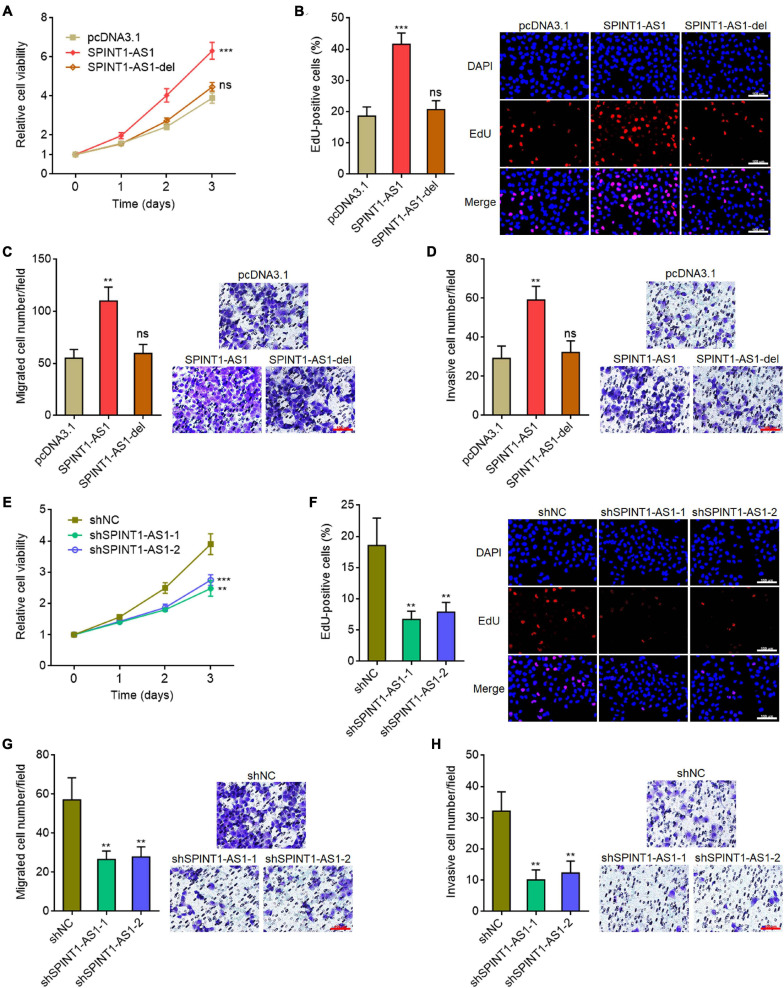
SPINT1-AS1 drove cervical cancer cellular proliferation, migration, and invasion in a miR-214 dependent manner. **(A)** Cell viabilities of HeLa cells with SPINT1-AS1 or 5′ 70 nucleotides deleted SPINT1-AS1 (SPINT1-AS1-del) stable overexpression were detected using Glo cell viability assay. **(B)** Cell proliferation of HeLa cells with SPINT1-AS1 or SPINT1-AS1-del stable overexpression was evaluated using EdU staining. Scale bars, 100 μm. **(C)** Cell migration of HeLa cells with SPINT1-AS1 or SPINT1-AS1-del stable overexpression was evaluated using transwell migration assay. Scale bars, 100 μm. **(D)** Cell invasion of HeLa cells with SPINT1-AS1 or SPINT1-AS1-del stable overexpression was evaluated by transwell invasion assay. Scale bars, 100 μm. **(E)** Cell viabilities of HeLa cells with SPINT1-AS1 stable silencing were detected by Glo cell viability assay. **(F)** Cell proliferation of HeLa cells with SPINT1-AS1 stable silencing was evaluated by EdU staining. Scale bars, 100 μm. **(G)** Cell migration of HeLa cells with SPINT1-AS1 stable silencing was evaluated by transwell migration assay. Scale bars, 100 μm. **(H)** Cell invasion of HeLa cells with SPINT1-AS1 stable silencing was detected by transwell invasion assay. Scale bars, 100 μm. Results are shown as mean ± SD based on three independent experiments. **P < 0.01, ***P < 0.001, ns, not significant, by one-way ANOVA followed by Dunnett′s multiple comparisons test.

miR-214 was frequently reported to be a tumor suppressive miRNA (Wu et al., 2018; Karimkhanloo et al., 2020). Here, we found that SPINT1-AS1 and miR-214 reciprocally repressed each other. Thus, we further investigated the potential roles of SPINT1-AS1 in cervical cancer. Glo cell viability assays demonstrated that overexpression of SPINT1-AS1 remarkably increased cell viability of HeLa cells, which was blocked by the deletion of DNM3OS binding site on SPINT1-AS1 (Figure 4A). EdU staining assays also demonstrated that overexpression of SPINT1-AS1 promoted cell proliferation of HeLa cells, which was abolished by the deletion of DNM3OS binding site on SPINT1-AS1 (Figure 4B). Transwell migration and invasion assays demonstrated that overexpression of SPINT1-AS1 promoted cell migration and invasion of HeLa cells, which were both abolished by the deletion of DNM3OS binding site on SPINT1-AS1 (Figures 4C,D). The roles of SPINT1-AS1 overexpression in driving cell proliferation, migration, and invasion were also verified in SiHa cells (Supplementary Figures 3A–D). The effects of SPINT1-AS1 silencing on cervical cell proliferation, migration, and invasion were further investigated using the same experiments. SPINT1-AS1 silencing repressed cell proliferation, migration, and invasion of HeLa cells (Figures 4E–H). The suppressive roles of SPINT1-AS1 silencing in cell proliferation, migration, and invasion were also verified in SiHa cells (Supplementary Figures 3E–H).

### SPINT1-AS1 Drove Cervical Cancer Tumorigenesis

**FIGURE 5:**
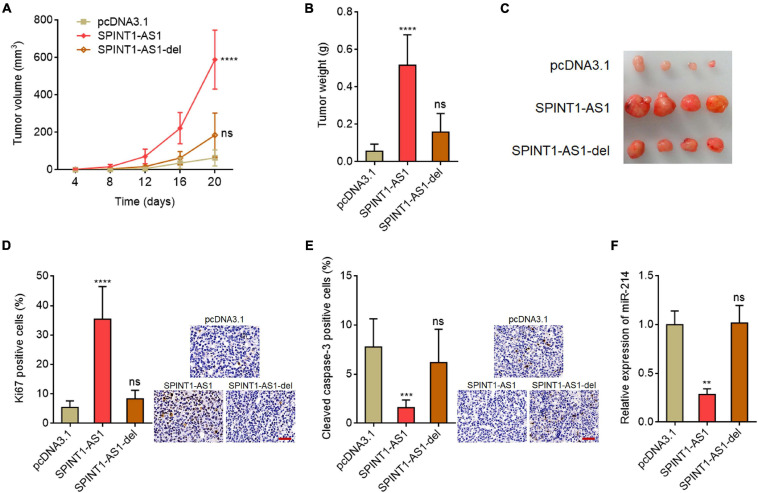
SPINT1-AS1 drove cervical cancer tumorigenesis *in vivo* in a miR-214 dependent manner. **(A)** HeLa cells with SPINT1-AS1 or 5′ 70 nucleotides deleted SPINT1-AS1 (SPINT1-AS1-del) stable overexpression were subcutaneously inoculated into nude mice. Tumor volumes were detected every 4 days. **(B)** Subcutaneous tumors were excised and weighed at the 20th day after inoculation. **(C)** Representative photo of subcutaneous tumors excised at the 20th day after inoculation. **(D)** Ki67 IHC staining was performed using the excised subcutaneous tumors. Scale bars, 50 μm. **(E)** Cleaved caspase-3 IHC staining was performed using the excised subcutaneous tumors. Scale bars, 50 μm. **(F)** miR-214 expressions in the excised subcutaneous tumors was detected by qRT-PCR. Results are shown as mean ± SD based on *n* = 7 mice in each group. **P < 0.01, ***P < 0.001, ****P < 0.0001, ns, not significant, by one-way ANOVA followed by Dunnett’s multiple comparisons test.

To investigate the potential biological roles of SPINT1-AS1 *in vivo*, full-length SPINT1-AS1 or 5′ 70 nucleotides deleted SPINT1-AS1 stably overexpressed and control HeLa cells were subcutaneously injected into nude mice. Subcutaneous tumor volumes were detected every 4 days. The results demonstrated that the tumors formed by full-length SPINT1-AS1 overexpressed HeLa cells grew significantly faster than control HeLa cells, which was reversed by the deletion of DNM3OS binding site on SPINT1-AS1 (Figure 5A). At the 20th day after inoculation, the subcutaneous tumors were resected and weighed. As demonstrated in Figures 5B,C, full-length SPINT1-AS1 overexpressed HeLa cells formed significantly heavier and larger tumors than control HeLa cells, which was largely reversed by the deletion of DNM3OS binding site on SPINT1-AS1. In addition, proliferation marker Ki67 IHC staining demonstrated that subcutaneous tumors formed by full-length SPINT1-AS1 overexpressed HeLa cells had increased Ki67 positive and proliferative cells compared to control HeLa cells, which was largely reversed by the deletion of DNM3OS binding site on SPINT1-AS1 (Figure 5D). Apoptosis marker cleaved caspase-3 IHC staining demonstrated that subcutaneous tumors formed by full-length SPINT1-AS1 overexpressed HeLa cells had less cleaved caspase-3 positive and apoptotic cells than control HeLa cells, which was largely reversed by the deletion of DNM3OS binding site on SPINT1-AS1 (Figure 5E). Consistent with the *in vitro* results, miR-214 was significantly downregulated in subcutaneous tumors formed by full-length SPINT1-AS1 overexpressed HeLa cells, but not in subcutaneous tumors formed by HeLa cells with DNM3OS binding site deleted SPINT1-AS1 overexpression (Figure 5F).

### SPINT1-AS1 Activated Wnt/β-Catenin Signaling via Repressing miR-214

**FIGURE 6:**
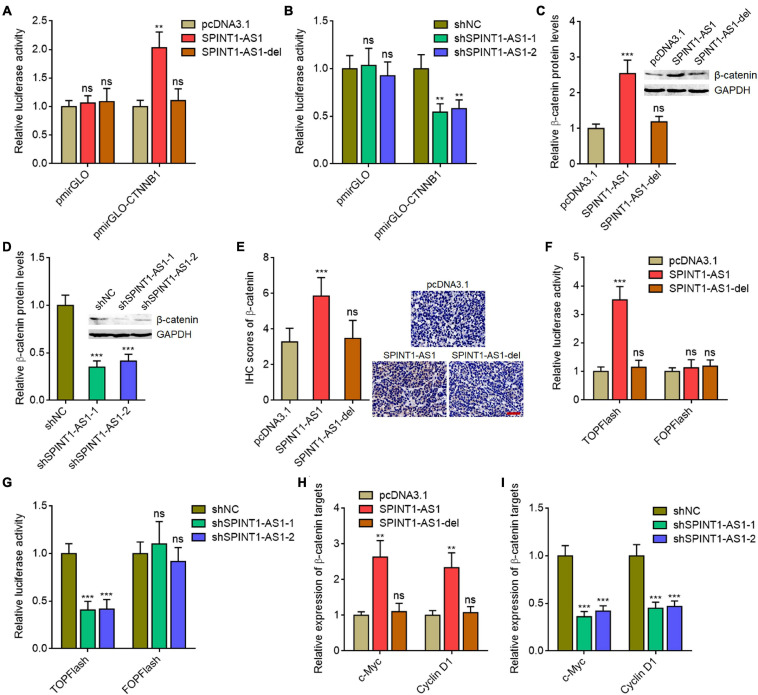
SPINT1-AS1 activated Wnt/β-catenin signaling in a miR-214 dependent manner. **(A)** Dual luciferase reporter assays in HeLa cells with SPINT1-AS1 or 5′ 70 nucleotides deleted SPINT1-AS1 (SPINT1-AS1-del) stable overexpression after transfection of luciferase reporter containing nothing or CTNNB1 3′-UTR. Data are shown as the relative ratio of firefly luciferase activity to renilla luciferase activity. **(B)** Dual luciferase reporter assays in HeLa cells with SPINT1-AS1 stable silencing after transfection of luciferase reporter containing nothing or CTNNB1 3′-UTR. Data are shown as the relative ratio of firefly luciferase activity to renilla luciferase activity. **(C)** β-catenin protein levels in HeLa cells with SPINT1-AS1 or SPINT1-AS1-del stable overexpression were detected by western blot. **(D)** β-catenin protein levels in HeLa cells with SPINT1-AS1 stable silencing were detected by western blot. **(E)** β-catenin IHC staining was performed using the excised subcutaneous tumors. **(F)** Dual luciferase reporter assays in HeLa cells with SPINT1-AS1 or SPINT1-AS1-del stable overexpression after transfection of TOPFlash or FOPFlash. Data are shown as the relative ratio of firefly luciferase activity to renilla luciferase activity. **(G)** Dual luciferase reporter assays in HeLa cells with SPINT1-AS1 stable silencing after transfection of TOPFlash or FOPFlash. Data are shown as the relative ratio of firefly luciferase activity to renilla luciferase activity. **(H)** β-catenin targets c-Myc and cyclin D1 expressions in HeLa cells with SPINT1-AS1 or SPINT1-AS1-del stable overexpression were detected by qRT-PCR. **(I)** β-catenin targets c-Myc and cyclin D1 expressions in HeLa cells with SPINT1-AS1 stable silencing was detected by qRT-PCR. Results are shown as mean ± SD based on three independent experiments. **P < 0.01, ***P < 0.001, ns, not significant, by one-way ANOVA followed by Dunnett′s multiple comparisons test.

The critical oncogenic protein β-catenin was a well-known miR-214 downstream target (Wu et al., 2018; Karimkhanloo et al., 2020). Given that SPINT1-AS1 exerts oncogenic roles in cervical cancer via binding DNM3OS and repressing miR-214 biogenesis, we further explored the potential roles of SPINT1-AS1 on Wnt/β-catenin signaling. CTNNB1 (encoding β-catenin) 3′-UTR sequences containing miR-214 binding site were inserted into the pmirGLO reporter. As expected, overexpression of full-length SPINT1-AS1, but not DNM3OS binding site deleted SPINT1-AS1 increased the luciferase activity of pmirGLO reporter containing CTNNB1 3′-UTR (Figure 6A). SPINT1-AS1 silencing reduced the luciferase activity of pmirGLO reporter containing CTNNB1 3′-UTR (Figure 6B). Western blot assays demonstrated that β-catenin was increased in SPINT1-AS1 overexpressed HeLa cells, but not HeLa cells with overexpression of DNM3OS binding site deleted SPINT1-AS1 (Figure 6C). Conversely, β-catenin was reduced in SPINT1-AS1 silenced HeLa cells (Figure 6D). Furthermore, β-catenin IHC staining demonstrated that subcutaneous tumors formed by full-length SPINT1-AS1 overexpressed HeLa cells had increased β-catenin expression compared with those formed by control HeLa cells, which was largely reversed by the deletion of DNM3OS binding site on SPINT1-AS1 (Figure 6E). To evaluate the influences of SPINT1-AS1 on Wnt/β-catenin signaling, β-catenin reporter TOPFlash or control reporter FOPFlash with mutated TCF/LEF binding sites was transfected into HeLa cells with overexpression of SPINT1-AS1 or DNM3OS binding site deleted SPINT1-AS1. Dual luciferase reporter assays revealed that SPINT1-AS1 overexpression upregulated the luciferase activity of TOPFlash, but not FOPFlash (Figure 6F). Deletion of DNM3OS binding site on SPINT1-AS1 abolished the increase of TOPFlash luciferase activity (Figure 6F). Furthermore, TOPFlash or FOPFlash was transfected into SPINT1-AS1 silenced HeLa cells. The results demonstrated that SPINT1-AS1 silencing reduced the luciferase activity of TOPFlash, but not FOPFlash (Figure 6G). Next, the expressions of Wnt/β-catenin targets c-Myc and cyclin D1 in SPINT1-AS1 overexpressed and silenced HeLa cells were detected. The results demonstrated that c-Myc and cyclin D1 were upregulated in HeLa cells with SPINT1-AS1 overexpression, but not with overexpression of DNM3OS binding site deleted SPINT1-AS1 (Figure 6H). Conversely, c-Myc and cyclin D1 were decreased in HeLa cells with SPINT1-AS1 silencing (Figure 6I). Collectively, these findings suggested that SPINT1-AS1 activated Wnt/β-catenin signaling in a miR-214 dependent manner.

### Overexpression of miR-214 or Inhibition of Wnt/β-Catenin Signaling Reversed the Oncogenic Roles of SPINT1-AS1 in Cervical Cancer

**FIGURE 7:**
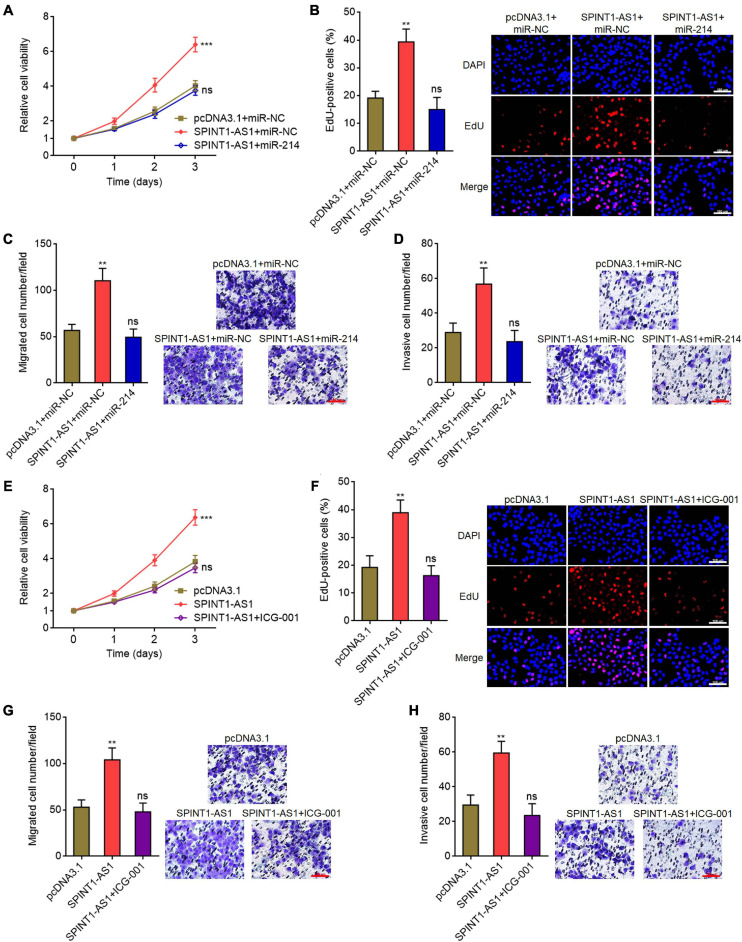
Overexpression of miR-214 or inhibition of Wnt/β-catenin signaling attenuated the oncogenic roles of SPINT1-AS1 in cervical cancer. **(A)** Cell viabilities of HeLa cells with SPINT1-AS1 and miR-214 overexpression were detected using Glo cell viability assay. **(B)** Cell proliferation of HeLa cells with SPINT1-AS1 and miR-214 overexpression was evaluated using EdU staining. Scale bars, 100 μm. **(C)** Cell migration of HeLa cells with SPINT1-AS1 and miR-214 overexpression was evaluated using transwell migration assay. Scale bars, 100 μm. **(D)** Cell invasion of HeLa cells with SPINT1-AS1 and miR-214 overexpression was detected by transwell invasion assay. Scale bars, 100 μm. **(E)** Cell viabilities of HeLa cells with SPINT1-AS1 overexpression after treatment with 20μM ICG-001 were detected by Glo cell viability assay. **(F)** Cell proliferation of HeLa cells with SPINT1-AS1 overexpression after treatment with 20μM ICG-001 was evaluated using EdU staining. Scale bars, 100 μm. **(G)** Cell migration of HeLa cells with SPINT1-AS1 overexpression after treatment with 20μM ICG-001 was evaluated by transwell migration assay. Scale bars, 100 μm. **(H)** Cell invasion of HeLa cells with SPINT1-AS1 overexpression after treatment with 20μM ICG-001 was detected by transwell invasion assay. Scale bars, 100 μm. Results are shown as mean ± SD based on three independent experiments. **P < 0.01, ***P < 0.001, ns, not significant, by one-way ANOVA followed by Dunnett’s multiple comparisons test.

To investigate whether the repression of miR-214 and activation of Wnt/β-catenin signaling mediated the oncogenic roles of SPINT1-AS1 in cervical cancer, we rescued miR-214 expression in SPINT1-AS1 overexpressed HeLa cells and then detected cell proliferation, migration, and invasion. Glo cell viability and EdU staining assays demonstrated that miR-214 overexpression abolished the pro-proliferative roles of SPINT1-AS1 (Figures 7A,B). Transwell migration assays demonstrated that miR-214 overexpression reversed the pro-migratory roles of SPINT1-AS1 (Figure 7C). Transwell invasion assays demonstrated that miR-214 overexpression abolished the pro-invasive roles of SPINT1-AS1 (Figure 7D). In addition, SPINT1-AS1 overexpressed HeLa cells were treated with 20μM Wnt/β-catenin signaling inhibitor ICG-001. Glo cell viability and EdU staining experiments demonstrated that ICG-001 treatment also abolished the pro-proliferative roles of SPINT1-AS1 (Figures 7E,F). Transwell migration and invasion assays demonstrated that ICG-001 treatment also abolished the pro-migratory and pro-invasive roles of SPINT1-AS1 (Figures 7G,H). Therefore, these findings suggested that the roles of SPINT1-AS1 in cervical cancer are dependent on the inhibition of miR-214 and activation of Wnt/β-catenin signaling.

## Discussion

Here, we identified a novel cervical cancer-associated lncRNA SPINT1-AS1. *SPINT1-AS1* is located in chromosome 15q15.1, which has three exons. Previous reports about the clinical relevance of SPINT-AS1 in cancers are inconsistent. SPINT1-AS1 was reported to be associated with good prognosis of renal clear cell carcinoma (Qi-Dong et al., 2020). SPINT1-AS1 was also reported to predict poor prognosis in colorectal cancer (Li et al., 2018). Functionally, SPINT1-AS1 was reported to promote breast cancer proliferation and metastasis (Zhou et al., 2021). SPINT1-AS1 knockdown promoted EGFR inhibitor lapatinib resistance in NCI-N87 and MCF7 cells (Xiang et al., 2019). Different clinical relevancies and roles of SPINT1-AS1 in different cancers suggested that the involvement of SPINT1-AS1 in cancers may be caner-specific.

We first revealed that SPINT1-AS1 is upregulated in cervical cancer. Increased SPINT1-AS1 levels in cervical cancer tissues are correlated with advanced stages and poor prognosis of cervical cancer patients. Functionally, we revealed that SPINT1-AS1 has oncogenic roles in cervical cancer. Ectopic expression of SPINT1-AS1 drives cervical cancer cellular proliferation, migration, and invasion *in vitro*, and also cervical cancer tumorigenesis *in vivo*. SPINT1-AS1 silencing has opposite effects in cervical cancer. Thus, our findings suggested SPINT1-AS1 as a cervical cancer-associated oncogenic lncRNA, which might be a potential prognostic biomarker and therapeutic target for cervical cancer.

Given the critical roles of miRNAs in cancers, the factors which modulate the expressions and/or functions of miRNAs may also have important roles in cancers. Many previous reports demonstrated that lncRNAs exert their oncogenic or tumor suppressive roles through competitively binding common miRNAs, leading to the relief of the repressive roles of miRNAs on miRNAs’ targets (Yuan et al., 2014; Song et al., 2019). lncRNA-ATB promoted hepatocellular carcinoma metastasis via sponging miR-200s (Yuan et al., 2014). Our previous study also found that lncRNA LINC01535 promoted cervical cancer progression via sponging miR-214 (Song et al., 2019). Here, we identified another mechanism of action of lncRNA, which is modulating miRNA biogenesis. Classically, primary miRNA (pri-miRNA) transcripts are cleaved by Microprocessor including DROSHA and DGCR8 to generate pre-miRNAs, which are further cleaved by DICER to yield mature miRNAs. Here, we demonstrated that SPINT1-AS1 directly binds DNM3OS, which is the primary transcript of miR-214 and gives rise to mature miR-214. The binding of SPINT1-AS1 to DNM3OS represses the binding of Microprocessor to DNM3OS, leading to the repression of DNM3OS cleavage and miR-214 biogenesis. Thus, our findings provided a relative novel functional manner of lncRNA on miRNAs, which is different from the frequently reported competitive sponging of miRNAs. The contributions of lncRNAs to miRNAs biogenesis are gradually being revealed. Wu et al. (2020) reported that lncRNA CYTOR represses miR-873-5p biogenesis via repressing the recruitment of DICER and TRBP to pre-miR-873 and further the cleavage of pre-miR-873. Zhang et al. (2021) reported that lncRNA HOTAIRM1 represses miR-144 biogenesis via reducing pre-miR-144 stability and blocking the processing of pri-miR-144 by DROSHA. Given the multiple processes of miRNAs biogenesis, lncRNAs may exert various mechanisms on different processes of miRNAs biogenesis, which need further studies.

Except the repressive roles of SPINT1-AS1 on miR-214 biogenesis, we also identified SPINT1-AS1 as a direct miR-214 target. Therefore, SPINT-AS1 and miR-214 forms a feedback loop. The reciprocal repressive roles between SPINT1-AS1 and miR-214 further induces the loss of equilibrium of SPINT1-AS1 and miR-214 expressions in cervical cancer, which may drive cervical cancer progression. Through repressing miR-214, SPINT1-AS1 upregulates the expression of β-catenin and further activates Wnt/β-catenin signaling. The 5′ 70 nucleotides of SPINT1-AS1, which mediates the interaction between SPINT1-AS1 and DNM3OS, are also responsible for the downregulation of miR-214 level, the upregulation of β-catenin, Wnt/β-catenin signaling activation, and the oncogenic roles of SPINT1-AS1 in cervical cancer. Furthermore, functional rescue assays also demonstrated that miR-214 and Wnt/β-catenin signaling mediate the oncogenic roles of SPINT1-AS1 in cervical cancer.

## Conclusion

**FIGURE 8:**
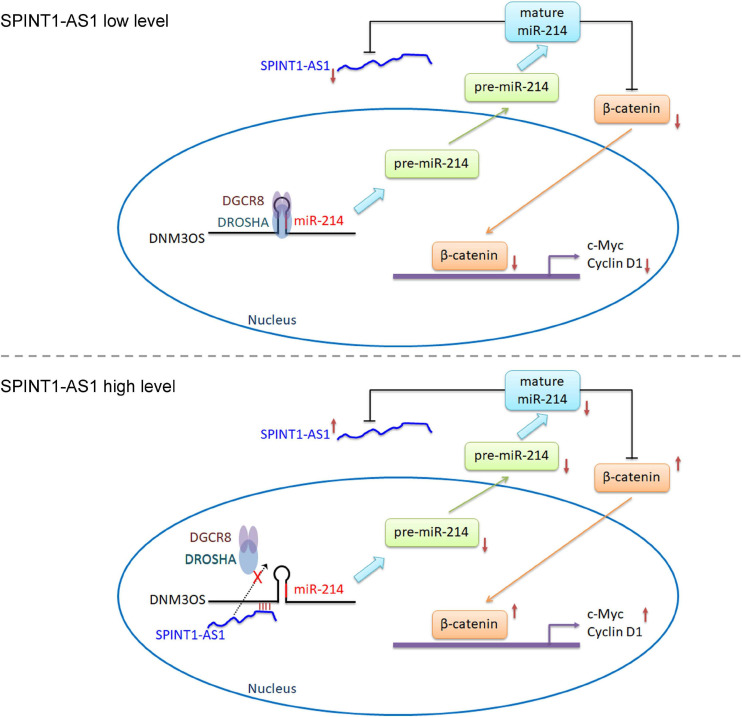
Schematic diagram of the reciprocal repression between SPINT1-AS1 and miR-214.

Our findings identified an upregulated and poor prognosis-related lncRNA, SPINT1-AS1, in cervical cancer. SPINT1-AS1 is a downstream target of miR-214. Furthermore, SPINT1-AS1 directly binds DNM3OS and represses the binding of DROSHA and DGCR8 to DNM3OS, leading to the suppression of DNM3OS cleavage and miR-214 biogenesis. Thus, SPINT1-AS1 and miR-214 form a double negative feedback loop. Via suppressing miR-214 biogenesis, SPINT1-AS1 upregulates β-catenin and activates Wnt/β-catenin signaling (Figure 8). Through modulating miR-214/Wnt/β-catenin signaling axis, SPINT1-AS1 drives cervical cancer cellular proliferation, migration, and invasion *in vitro*, and tumorigenesis *in vivo*. These findings provide a novel understanding of the pathological mechanism of cervical cancer, which may represent potential therapeutic option for cervical cancer.

## Data Availability Statement

The raw data supporting the conclusions of this article will be made available by the authors, without undue reservation.

## Ethics Statement

The studies involving human participants were reviewed and approved by the Ethics Committee of Xuzhou Maternity and Child Health Care Hospital. The patients/participants provided their written informed consent to participate in this study. The animal study was reviewed and approved by the Ethics Committee of Xuzhou Maternity and Child Health Care Hospital.

## Author Contributions

HS designed, supervised the study, and wrote the manuscript. HS, YL, HL, XJ, and LL performed the experiments. HS and YL collected and analyzed the data. All authors approved the final version of the manuscript.

## Conflict of Interest

LL was employed by company Shanghai Lichun Biotechnology Co., Ltd. The remaining authors declare that the research was conducted in the absence of any commercial or financial relationships that could be construed as a potential conflict of interest.

## Footnotes

1 http://starbase.sysu.edu.cn/

2 http://gepia.cancer-pku.cn/

3 http://lilab.research.bcm.edu/cpat/index.php

4 http://cpc2.gao-lab.org/

5 http://www.csbio.sjtu.edu.cn/bioinf/lncLocator/

6 http://rna.informatik.uni-freiburg.de/IntaRNA/Input.jsp
