# Supplementary material for: SPINT1-AS1 Drives Cervical Cancer Progression via Repressing miR-214 Biogenesis
Source: Front Cell Dev Biol. 2021 Jul 19;9:691140. doi: 10.3389/fcell.2021.691140 (PMC8326843; doi:10.3389/fcell.2021.691140)
Supplement: Supplementary file 1 [file Table_1.DOCX]

## Supplementary figure legends

**Figure S1.** The characteristics of SPINT1-AS1. **(A)** SPINT1-AS1 expression in 306 cervical squamous cell carcinoma and endocervical adenocarcinoma (CESC) tissues and 13 normal cervical tissues from The Cancer Genome Atlas (TCGA) and the Genotype-Tissue Expression (GTEx) projects analyzed by GEPIA (Gene Expression Profiling Interactive Analysis). **(B)** The coding potential of SPINT1-AS1 was evaluated by the Coding Potential Assessment Tool (CPAT). **(C)** The coding potential of SPINT1-AS1 was evaluated by the Coding Potential Calculator (CPC). **(D)** Subcellular location of SPINT1-AS1 was analyzed by lncLocator. **(E)** Subcellular location of SPINT1-AS1 in HeLa cells was detected using subcellular fraction, followed by qRT-PCR. GAPDH and U6 were used as cytoplasmic and nucleic controls, respectively. Results are shown as mean ± SD based on three independent experiments.

**Figure S2.** SPINT1-AS1 repressed miR-214 biogenesis in SiHa cells. **(A)** SPINT1-AS1 and miR-214 expressions in SiHa cells with SPINT1-AS1 stable overexpression were detected by qRT-PCR. **(B)** SPINT1-AS1 and miR-214 expressions in SiHa cells with SPINT1-AS1 stable silencing were detected by qRT-PCR. **(C)** DNM3OS expression in SiHa cells with SPINT1-AS1 stable overexpression were detected by qRT-PCR. **(D)** DNM3OS expression in SiHa cells with SPINT1-AS1 stable silencing were detected by qRT-PCR. **(E)** miR-199a and miR-3120 expressions in HeLa cells with SPINT1-AS1 stable overexpression were detected by qRT-PCR. **(F)** miR-199a and miR-3120 expressions in HeLa cells with SPINT1-AS1 stable silencing were detected by qRT-PCR. Results are shown as mean ± SD based on three independent experiments. ***P* < 0.01, ****P* < 0.001, *****P* < 0.0001, ns, not significant, by Student's *t* test (**A**, **C, E**) or one-way ANOVA followed by Dunnett's multiple comparisons test (**B**, **D, F**).

**Figure S3.** SPINT1-AS1 drove SiHa cell proliferation, migration, and invasion. **(A)** Cell viabilities of SiHa cells with SPINT1-AS1 stable overexpression were detected by Glo cell viability assay. **(B)** Cell proliferation of SiHa cells with SPINT1-AS1 stable overexpression was detected by EdU staining. Scale bars, 100 µm. **(C)** Cell migration of SiHa cells with SPINT1-AS1 stable overexpression was detected by transwell migration assay. Scale bars, 100 µm. **(D)** Cell invasion of SiHa cells with SPINT1-AS1 stable overexpression was detected by transwell invasion assay. Scale bars, 100 µm. **(E)** Cell viabilities of SiHa cells with SPINT1-AS1 stable silencing were detected by Glo cell viability assay. **(F)** Cell proliferation of SiHa cells with SPINT1-AS1 stable silencing was detected by EdU staining. Scale bars, 100 µm. **(G)** Cell migration of SiHa cells with SPINT1-AS1 stable silencing was detected by transwell migration assay. Scale bars, 100 µm. **(H)** Cell invasion of SiHa cells with SPINT1-AS1 stable silencing was detected by transwell invasion assay. Scale bars, 100 µm. Results are shown as mean ± SD based on three independent experiments. **P* < 0.05, ***P* < 0.01 by Student's *t* test (**A**-**D**) or one-way ANOVA followed by Dunnett's multiple comparisons test (**E**-**H**).
