# Supplementary figures and images for: SPINT1-AS1 Drives Cervical Cancer Progression via Repressing miR-214 Biogenesis
Source: Front Cell Dev Biol. 2021 Jul 19;9:691140. doi: 10.3389/fcell.2021.691140 (PMC8326843; doi:10.3389/fcell.2021.691140)

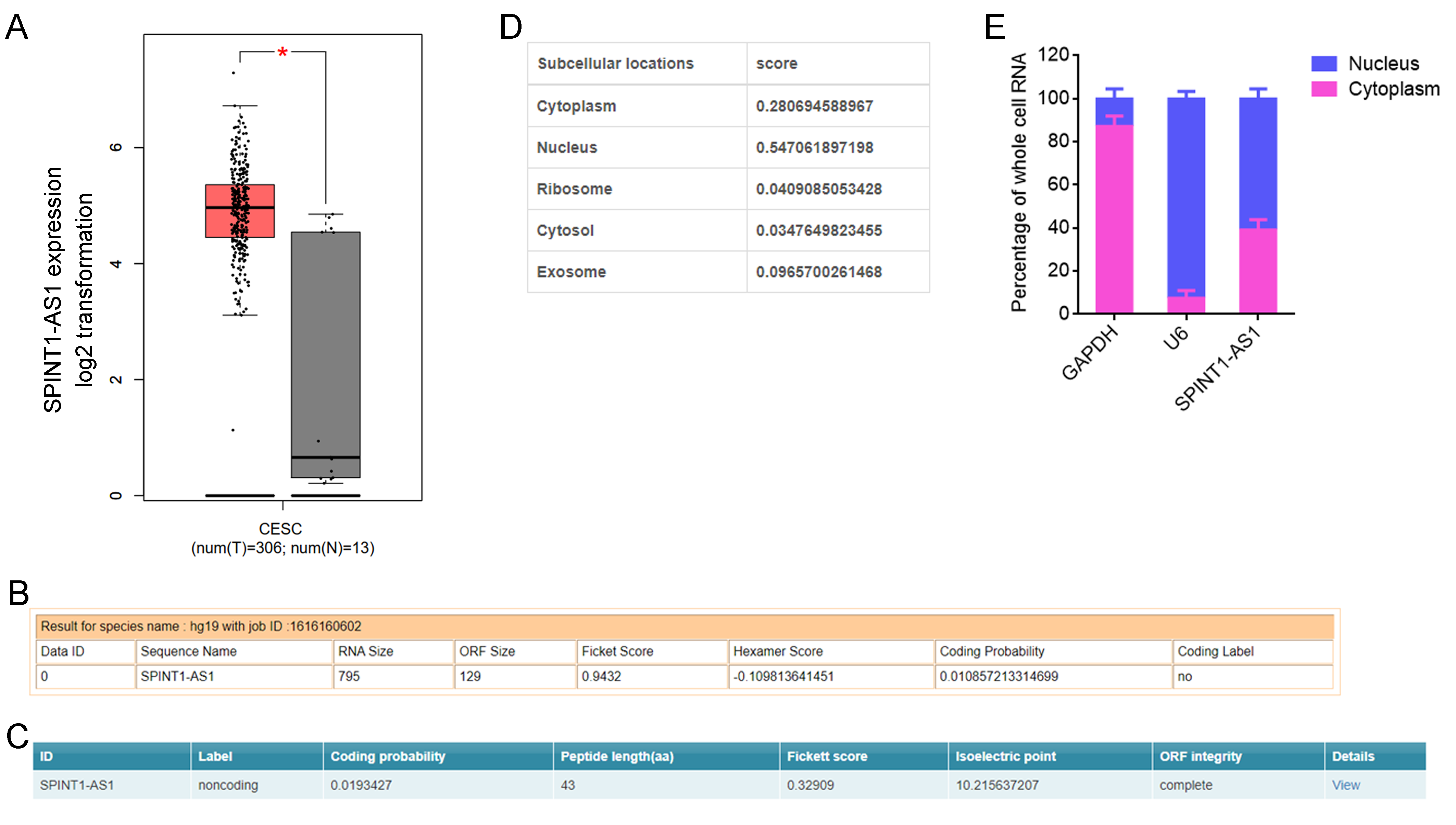

Supplement: Supplementary file 2 [file Image_1.TIF]

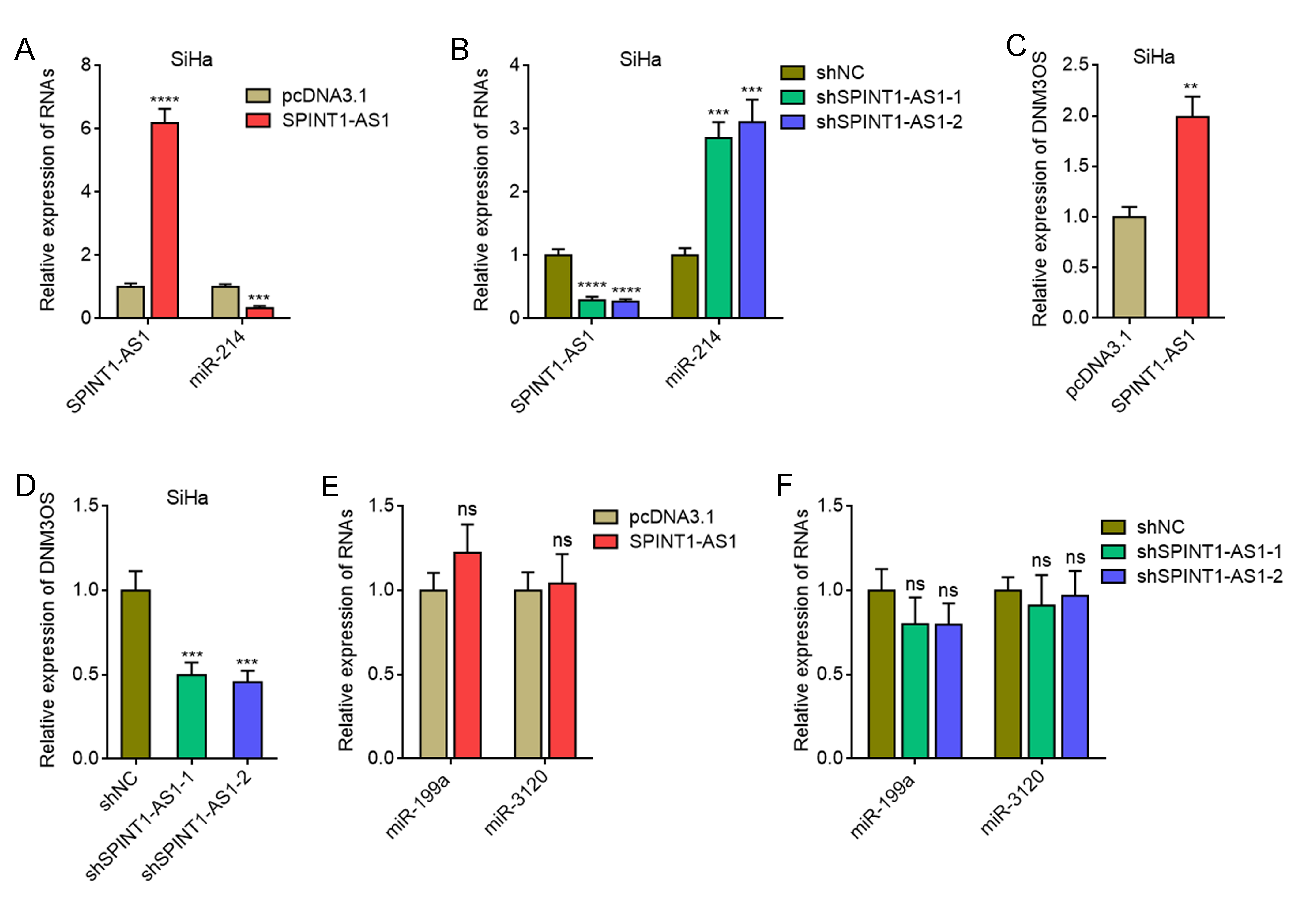

Supplement: Supplementary file 3 [file Image_2.TIF]

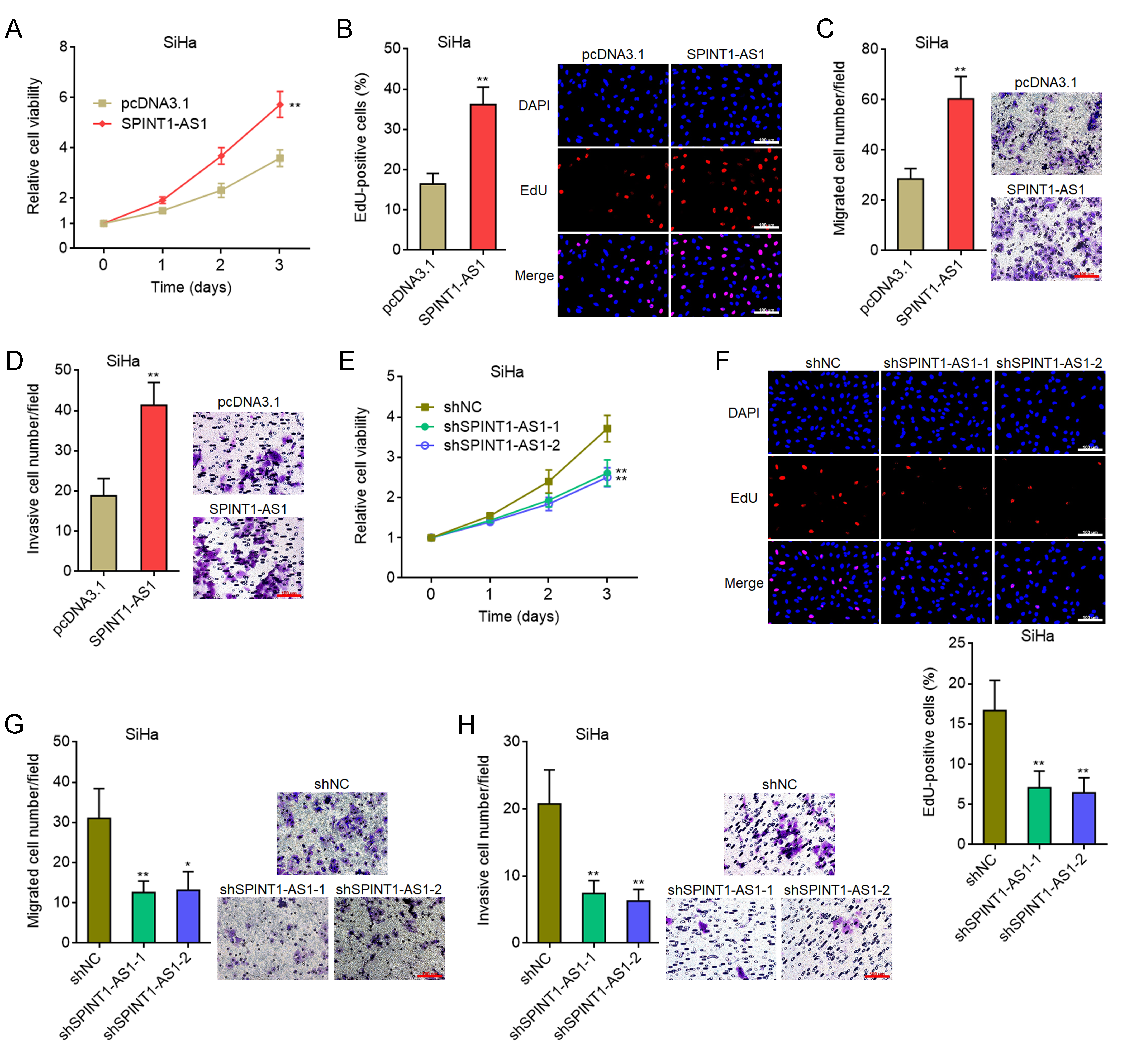

Supplement: Supplementary file 4 [file Image_3.TIF]
